# Tetra­aqua­bis­(3,5-dinitro­benzoato-κ*O* ^1^)magnesium tetra­hydrate

**DOI:** 10.1107/S160053681300682X

**Published:** 2013-03-16

**Authors:** Graham Smith

**Affiliations:** a Science and Engineering Faculty, Queensland University of Technology, GPO Box 2434, Brisbane, Queensland 4001, Australia

## Abstract

In the structure of the title compound, [Mg(C_7_H_3_N_2_O_6_)_2_(H_2_O)_4_]·4H_2_O, the slightly distorted octa­hedral MgO_6_ coord­in­ation polyhedron comprises two *trans*-related carboxyl­ate O-atom donors from mononodentate 3,5-dinitro­benzoate ligands, and four water mol­ecules. The coordinating water mol­ecules and the four water mol­ecules of solvation give both intra- and inter-unit O—H⋯O hydrogen-bonding inter­actions with carboxyl­ate, water and nitro O-atom acceptors, forming a three-dimensional structure.

## Related literature

For the structures of some magnesium complexes with nitro-substituted benzoic acids, see: Morgant *et al.* (2006 ▶); Srinivasan *et al.* (2007 ▶, 2011 ▶); Arlin *et al.* (2011 ▶).
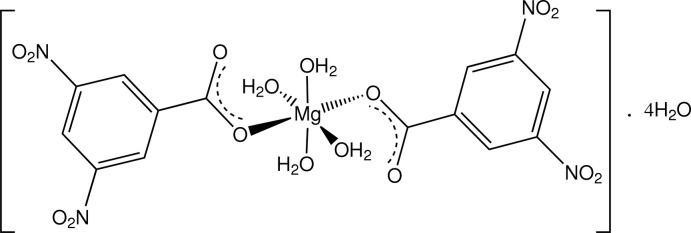


## Experimental

### 

#### Crystal data

[Mg(C_7_H_3_N_2_O_6_)_2_(H_2_O)_4_]·4H_2_O
*M* *_r_* = 590.67
Triclinic, 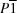

*a* = 7.1748 (3) Å
*b* = 11.7299 (6) Å
*c* = 15.0103 (7) Å
α = 103.224 (4)°
β = 98.569 (4)°
γ = 92.181 (4)°
*V* = 1212.62 (10) Å^3^
*Z* = 2
Mo *K*α radiation
μ = 0.18 mm^−1^
*T* = 200 K
0.32 × 0.22 × 0.10 mm

#### Data collection

Oxford Diffraction Gemini-S CCD-detector diffractometer
Absorption correction: multi-scan (*CrysAlis PRO*; Agilent, 2012 ▶) *T*_min_ = 0.970, *T*_max_ = 0.980
15059 measured reflections
4764 independent reflections
3969 reflections with *I* > 2σ(*I*)
*R*_int_ = 0.027

#### Refinement

*R*[*F* ^2^ > 2σ(*F* ^2^)] = 0.036
*wR*(*F* ^2^) = 0.095
*S* = 0.94
4764 reflections
352 parameters
H-atom parameters constrained
Δρ_max_ = 0.29 e Å^−3^
Δρ_min_ = −0.20 e Å^−3^

### 

Data collection: *CrysAlis PRO* (Agilent, 2012 ▶); cell refinement: *CrysAlis PRO*; data reduction: *CrysAlis PRO*; program(s) used to solve structure: *SHELXS97* (Sheldrick, 2008 ▶); program(s) used to refine structure: *SHELXL97* (Sheldrick, 2008 ▶) within *WinGX* (Farrugia, 2012 ▶); molecular graphics: *PLATON* (Spek, 2009 ▶); software used to prepare material for publication: *PLATON*.

## Supplementary Material

Crystal structure: contains datablock(s) global, I. DOI: 10.1107/S160053681300682X/sj5305sup1.cif

Structure factors: contains datablock(s) I. DOI: 10.1107/S160053681300682X/sj5305Isup2.hkl

Additional supplementary materials:  crystallographic information; 3D view; checkCIF report

## Figures and Tables

**Table 1:** Selected bond lengths (Å)

Mg1—O1*W*	2.0929 (14)
Mg1—O2*W*	2.0732 (13)
Mg1—O3*W*	2.1024 (14)
Mg1—O4*W*	2.0804 (13)
Mg1—O11*A*	2.0304 (13)
Mg1—O11*B*	2.0237 (13)

**Table 2:** Hydrogen-bond geometry (Å, °)

*D*—H⋯*A*	*D*—H	H⋯*A*	*D*⋯*A*	*D*—H⋯*A*
O1*W*—H11*W*⋯O8*W*	0.91	1.79	2.700 (2)	179
O1*W*—H12*W*⋯O6*W* ^i^	0.88	1.93	2.7934 (19)	170
O2*W*—H21*W*⋯O12*A*	0.76	2.11	2.8001 (18)	152
O2*W*—H22*W*⋯O6*W*	0.87	1.87	2.7375 (18)	178
O3*W*—H31*W*⋯O7*W*	0.80	2.02	2.8213 (19)	170
O3*W*—H32*W*⋯O5*W* ^ii^	0.90	1.89	2.7722 (18)	170
O4*W*—H41*W*⋯O12*B*	0.80	2.00	2.7310 (18)	151
O4*W*—H42*W*⋯O5*W* ^iii^	0.83	1.97	2.7986 (18)	174
O5*W*—H51*W*⋯O7*W* ^iii^	0.86	2.11	2.9449 (19)	164
O5*W*—H52*W*⋯O1*W*	0.86	2.17	2.9702 (19)	155
O6*W*—H61*W*⋯O12*A* ^i^	0.86	2.00	2.8404 (19)	163
O6*W*—H62*W*⋯O3*W* ^iv^	0.86	2.14	2.9522 (19)	159
O7*W*—H71*W*⋯O12*B* ^v^	0.89	1.87	2.708 (2)	158
O7*W*—H72*W*⋯O32*B* ^vi^	0.86	2.50	3.236 (2)	145
O8*W*—H81*W*⋯O12*A* ^i^	0.90	1.99	2.7737 (19)	145

Symmetry codes: (i) 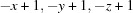; (ii) 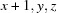; (iii) 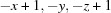; (iv) 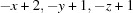; (v) 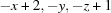; (vi) 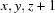.

**Fig. 1.**
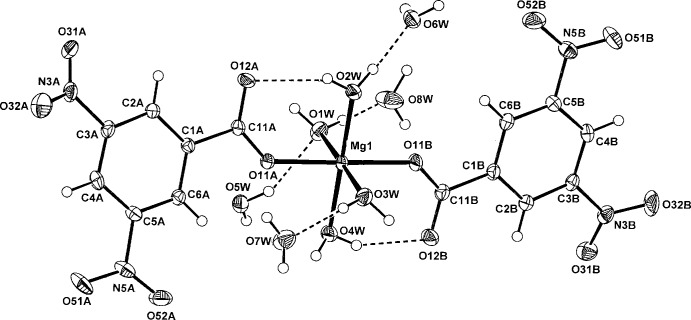
Molecular configuration and atom naming scheme for the title complex, with displacement ellipsoids drawn at the 50% probability level. Hydrogen bonds are shown as dashed lines.

**Fig. 2.**
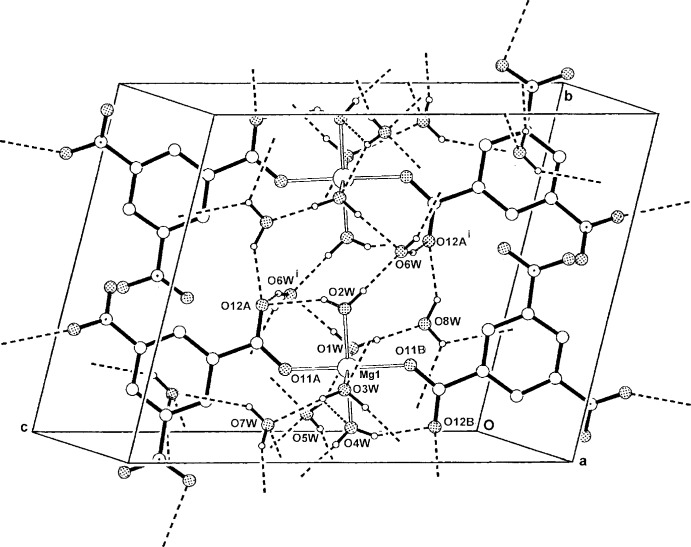
A perspective view of the title complex showing hydrogen-bonding interactions as dashed lines and with non-associative H-atoms omitted. For symmetry code (i), see Table 2.

## supplementary crystallographic information

### Comment

Magnesium complexes involving monoanionic nitro-substituted benzoate ligands (*L*) show both the common [Mg(H_2_O)_6_]^2+^ 2(*L*) form, *e.g.* a dihydrate: *L* = 4-nitrobenzoate (Srinivasan *et al.*, 2007; Arlin *et al.*, 2011), as well as examples in which the ligand is coordinated, *e.g.* [MgL(H_2_O)_5_] (*L*). HL. H_2_O (a complex acid adduct: *L* = 4-nitro-3-hydroxybenzoate) (Morgant *et al.*, 2006) and [Mg*L*_2_(H_2_O)_4_]: (*L* = 2-nitrobenzoate) (Srinivasan *et al.*, 2011; Arlin *et al.*, 2011). All known examples are monomeric and have essentially octahedral metal stereochemistry.

The title complex, [Mg(C_7_H_3_N_2_O_6_)_2_(H_2_O)_4_]. 4H_2_O was obtained from the reaction of 3,5-dinitrobenzoic acid acid with MgCO_3_ in aqueous ethanol and the structure is reported here. In this structure (Fig. 1), the slightly distorted octahedral MgO_6_ coordination polyhedron comprises two *trans*-related carboxyl O-atom donors from mononodentate 3,5-dinitrobenzoate ligands, and four water molecules [bond range Mg—O, 2.0237 (13)–2.1024 (14) Å (Table 1)]. The coordinated water molecules and the four water molecules of solvation give both intra- and inter-unit O—H···O hydrogen-bonding interactions with carboxyl, water and nitro O-atom acceptors (Table 2), giving a three-dimensional structure (Fig. 2).

In the present complex, the two 3,5-dinitrobenzoate ligands are conformationally similar, with the carboxyl groups lying essentially in the plane of the benzene ring [torsion angles C2—C1—C11—O11 = 178.01 (14)° (*A*) and 178.90 (14)° (*B*)]. The C5 nitro groups are variously rotated out of the benzene plane [torsion angles C2—C3—N3—O32 = 154.31 (17)° (*A*) and 159.03 (15)° (*B*): C4—C5—N5—O52 = 167.74 (15)° (*A*) and 163.06 (15)° (*B*)].

### Experimental

The title compound was synthesized by the addition of excess MgCO_3_ to 15 ml of a hot aqueous ethanolic solution (10:1) of 3,5-dinitrobenzoic acid (0.1 g). After completion of the reaction, the excess MgCO_3_ was removed by filtration and the solution was allowed to evaporate to partial dryness at room temperature, giving colourless plates of the title compound from which a specimen was cleaved for the X-ray analysis.

### Refinement

Hydrogen atoms on all water molecules were located by difference methods and both positional and isotropic displacement parameters were initially refined but these were then allowed to ride, with *U*_iso_(H) = 1.5*U*_eq_(O). Other H-atoms were included in the refinement at calculated positions [C—H = 0.93 Å] with *U*_iso_(H) = 1.2*U*_eq_(C) also using a riding-model approximation.

### Crystal data

**Table:**

[Mg(C_7_H_3_N_2_O_6_)_2_(H_2_O)_4_]·4H_2_O	*Z* = 2
*M**_r_* = 590.67	*F*(000) = 612
Triclinic, *P*1	*D*_x_ = 1.618 Mg m^−^^3^
Hall symbol: -P 1	Mo *K*α radiation, λ = 0.71073 Å
*a* = 7.1748 (3) Å	Cell parameters from 4480 reflections
*b* = 11.7299 (6) Å	θ = 3.3–28.8°
*c* = 15.0103 (7) Å	µ = 0.18 mm^−^^1^
α = 103.224 (4)°	*T* = 200 K
β = 98.569 (4)°	Plate, colourless
γ = 92.181 (4)°	0.32 × 0.22 × 0.10 mm
*V* = 1212.62 (10) Å^3^	

### Data collection

**Table:**

Oxford Diffraction Gemini-S CCD-detector diffractometer	4764 independent reflections
Radiation source: Enhance(Mo) X-ray source	3969 reflections with *I* > 2σ(*I*)
Graphite monochromator	*R*_int_ = 0.027
Detector resolution: 16.077 pixels mm^-1^	θ_max_ = 26.0°, θ_min_ = 3.3°
ω scans	*h* = −8→8
Absorption correction: multi-scan (*CrysAlis PRO*; Agilent, 2012)	*k* = −14→14
*T*_min_ = 0.970, *T*_max_ = 0.980	*l* = −18→18
15059 measured reflections	

### Refinement

**Table:**

Refinement on *F*^2^	Primary atom site location: structure-invariant direct methods
Least-squares matrix: full	Secondary atom site location: difference Fourier map
*R*[*F*^2^ > 2σ(*F*^2^)] = 0.036	Hydrogen site location: inferred from neighbouring sites
*wR*(*F*^2^) = 0.095	H-atom parameters constrained
*S* = 0.94	*w* = 1/[σ^2^(*F*_o_^2^) + (0.0457*P*)^2^ + 0.5437*P*] where *P* = (*F*_o_^2^ + 2*F*_c_^2^)/3
4764 reflections	(Δ/σ)_max_ < 0.001
352 parameters	Δρ_max_ = 0.29 e Å^−^^3^
0 restraints	Δρ_min_ = −0.20 e Å^−^^3^

### Special details

**Table:**

Geometry. Bond distances, angles *etc*. have been calculated using the rounded fractional coordinates. All su's are estimated from the variances of the (full) variance-covariance matrix. The cell e.s.d.'s are taken into account in the estimation of distances, angles and torsion angles
Refinement. Refinement of *F*^2^ against ALL reflections. The weighted *R*-factor *wR* and goodness of fit *S* are based on *F*^2^, conventional *R*-factors *R* are based on *F*, with *F* set to zero for negative *F*^2^. The threshold expression of *F*^2^ > σ(*F*^2^) is used only for calculating *R*-factors(gt) *etc*. and is not relevant to the choice of reflections for refinement. *R*-factors based on *F*^2^ are statistically about twice as large as those based on *F*, and *R*- factors based on ALL data will be even larger.

### Fractional atomic coordinates and isotropic or equivalent isotropic displacement parameters (Å^2^)

**Table:**

	*x*	*y*	*z*	*U*_iso_*/*U*_eq_	
Mg1	0.72688 (8)	0.24686 (5)	0.49442 (4)	0.0206 (2)	
O1W	0.45776 (18)	0.27809 (11)	0.43347 (9)	0.0315 (4)	
O2W	0.80173 (18)	0.42361 (10)	0.55275 (8)	0.0287 (4)	
O3W	0.99516 (17)	0.20820 (11)	0.55261 (9)	0.0289 (4)	
O4W	0.65142 (18)	0.06937 (10)	0.43663 (8)	0.0283 (4)	
O11A	0.62746 (18)	0.23370 (10)	0.61171 (8)	0.0255 (4)	
O11B	0.83217 (18)	0.26394 (10)	0.37996 (8)	0.0278 (4)	
O12A	0.5701 (2)	0.41362 (10)	0.68573 (9)	0.0318 (4)	
O12B	0.8071 (2)	0.08551 (11)	0.28425 (9)	0.0325 (4)	
O31A	0.31477 (19)	0.44416 (11)	0.97944 (9)	0.0340 (4)	
O31B	0.8787 (2)	0.06471 (11)	−0.04095 (9)	0.0351 (4)	
O32A	0.4359 (3)	0.33267 (14)	1.06560 (10)	0.0526 (6)	
O32B	1.0519 (2)	0.20264 (14)	−0.06886 (10)	0.0476 (5)	
O51A	0.5101 (2)	−0.07040 (12)	0.88966 (10)	0.0432 (5)	
O51B	1.0734 (2)	0.59134 (12)	0.13968 (10)	0.0420 (5)	
O52A	0.6406 (2)	−0.08279 (12)	0.76731 (10)	0.0429 (5)	
O52B	0.9300 (2)	0.60972 (12)	0.25868 (10)	0.0447 (5)	
N3A	0.3977 (2)	0.35844 (14)	0.99081 (10)	0.0303 (5)	
N3B	0.9615 (2)	0.16217 (13)	−0.01841 (10)	0.0273 (5)	
N5A	0.5629 (2)	−0.02866 (13)	0.82933 (11)	0.0300 (5)	
N5B	0.9901 (2)	0.55022 (13)	0.19201 (11)	0.0288 (5)	
C1A	0.5416 (2)	0.25645 (14)	0.75959 (11)	0.0191 (5)	
C1B	0.8897 (2)	0.25074 (15)	0.22763 (11)	0.0221 (5)	
C2A	0.4858 (2)	0.32878 (14)	0.83639 (11)	0.0215 (5)	
C2B	0.9050 (2)	0.18121 (15)	0.14109 (11)	0.0229 (5)	
C3A	0.4564 (2)	0.28082 (15)	0.90967 (11)	0.0235 (5)	
C3B	0.9496 (2)	0.23575 (15)	0.07369 (11)	0.0227 (5)	
C4A	0.4796 (2)	0.16471 (15)	0.91068 (12)	0.0255 (5)	
C4B	0.9805 (2)	0.35601 (15)	0.08781 (12)	0.0245 (5)	
C5A	0.5332 (2)	0.09582 (14)	0.83218 (12)	0.0226 (5)	
C5B	0.9603 (2)	0.42181 (15)	0.17419 (12)	0.0228 (5)	
C6A	0.5640 (2)	0.13877 (14)	0.75691 (11)	0.0211 (5)	
C6B	0.9152 (2)	0.37241 (15)	0.24432 (11)	0.0227 (5)	
C11A	0.5832 (2)	0.30567 (14)	0.67950 (11)	0.0211 (5)	
C11B	0.8402 (2)	0.19519 (15)	0.30354 (12)	0.0234 (5)	
O5W	0.21801 (18)	0.07011 (11)	0.44221 (9)	0.0295 (4)	
O6W	0.75569 (18)	0.58555 (11)	0.44724 (9)	0.0298 (4)	
O7W	1.0086 (2)	0.11013 (12)	0.70823 (9)	0.0413 (5)	
O8W	0.4051 (3)	0.34282 (12)	0.27062 (10)	0.0526 (6)	
H2A	0.46860	0.40750	0.83860	0.0260*	
H2B	0.88570	0.09990	0.12880	0.0270*	
H4A	0.46040	0.13470	0.96110	0.0310*	
H4B	1.01310	0.39060	0.04180	0.0290*	
H6A	0.59920	0.08960	0.70520	0.0250*	
H6B	0.90230	0.41970	0.30160	0.0270*	
H11W	0.43930	0.30080	0.37910	0.0470*	
H12W	0.37960	0.31880	0.46530	0.0470*	
H21W	0.73890	0.44440	0.58890	0.0430*	
H22W	0.79040	0.47460	0.51890	0.0430*	
H31W	1.01100	0.18470	0.59930	0.0430*	
H32W	1.05640	0.16380	0.51110	0.0430*	
H41W	0.69830	0.04970	0.39120	0.0420*	
H42W	0.68820	0.02370	0.46960	0.0420*	
H51W	0.16520	0.02280	0.39150	0.0440*	
H52W	0.30810	0.11320	0.43140	0.0440*	
H61W	0.66770	0.57420	0.39940	0.0450*	
H62W	0.84670	0.63140	0.44130	0.0450*	
H71W	1.07840	0.05610	0.72630	0.0620*	
H72W	1.00000	0.16100	0.75820	0.0620*	
H81W	0.42210	0.41470	0.26020	0.0790*	
H82W	0.40670	0.28650	0.22020	0.0790*	

### Atomic displacement parameters (Å^2^)

**Table:**

	*U*^11^	*U*^22^	*U*^33^	*U*^12^	*U*^13^	*U*^23^
Mg1	0.0281 (3)	0.0207 (3)	0.0153 (3)	0.0025 (2)	0.0077 (2)	0.0060 (2)
O1W	0.0356 (7)	0.0391 (8)	0.0244 (7)	0.0139 (6)	0.0088 (5)	0.0129 (6)
O2W	0.0430 (8)	0.0220 (6)	0.0251 (7)	0.0031 (5)	0.0129 (6)	0.0089 (5)
O3W	0.0328 (7)	0.0321 (7)	0.0250 (7)	0.0092 (5)	0.0088 (5)	0.0097 (6)
O4W	0.0399 (7)	0.0237 (6)	0.0228 (6)	0.0013 (5)	0.0099 (5)	0.0059 (5)
O11A	0.0404 (7)	0.0216 (6)	0.0179 (6)	0.0038 (5)	0.0136 (5)	0.0056 (5)
O11B	0.0415 (7)	0.0256 (6)	0.0190 (6)	0.0001 (5)	0.0136 (5)	0.0055 (5)
O12A	0.0574 (9)	0.0193 (6)	0.0236 (7)	0.0065 (6)	0.0152 (6)	0.0088 (5)
O12B	0.0526 (8)	0.0236 (7)	0.0247 (7)	0.0023 (6)	0.0136 (6)	0.0078 (5)
O31A	0.0372 (7)	0.0306 (7)	0.0344 (8)	0.0046 (6)	0.0177 (6)	0.0003 (6)
O31B	0.0460 (8)	0.0303 (7)	0.0251 (7)	0.0022 (6)	0.0021 (6)	0.0010 (6)
O32A	0.0864 (12)	0.0571 (10)	0.0185 (7)	0.0109 (9)	0.0189 (7)	0.0099 (7)
O32B	0.0645 (10)	0.0541 (10)	0.0262 (8)	−0.0070 (8)	0.0260 (7)	0.0035 (7)
O51A	0.0639 (10)	0.0333 (8)	0.0357 (8)	−0.0119 (7)	0.0008 (7)	0.0221 (7)
O51B	0.0491 (9)	0.0371 (8)	0.0434 (9)	−0.0119 (7)	0.0087 (7)	0.0187 (7)
O52A	0.0676 (10)	0.0223 (7)	0.0389 (8)	0.0090 (7)	0.0113 (7)	0.0047 (6)
O52B	0.0724 (11)	0.0252 (7)	0.0356 (8)	0.0060 (7)	0.0147 (7)	0.0014 (6)
N3A	0.0367 (9)	0.0340 (9)	0.0208 (8)	−0.0016 (7)	0.0129 (7)	0.0034 (7)
N3B	0.0321 (8)	0.0322 (9)	0.0179 (7)	0.0062 (7)	0.0059 (6)	0.0048 (7)
N5A	0.0414 (9)	0.0209 (8)	0.0271 (8)	−0.0037 (7)	−0.0023 (7)	0.0104 (7)
N5B	0.0337 (8)	0.0254 (8)	0.0264 (8)	−0.0017 (6)	−0.0012 (7)	0.0090 (7)
C1A	0.0215 (8)	0.0205 (8)	0.0159 (8)	0.0001 (6)	0.0035 (6)	0.0054 (7)
C1B	0.0234 (8)	0.0258 (9)	0.0193 (8)	0.0033 (7)	0.0076 (7)	0.0067 (7)
C2A	0.0260 (9)	0.0204 (8)	0.0188 (8)	0.0029 (7)	0.0053 (7)	0.0051 (7)
C2B	0.0267 (9)	0.0227 (9)	0.0203 (9)	0.0021 (7)	0.0073 (7)	0.0048 (7)
C3A	0.0269 (9)	0.0278 (9)	0.0160 (8)	0.0013 (7)	0.0077 (7)	0.0033 (7)
C3B	0.0242 (9)	0.0284 (9)	0.0160 (8)	0.0041 (7)	0.0064 (7)	0.0042 (7)
C4A	0.0298 (9)	0.0313 (10)	0.0178 (8)	−0.0032 (7)	0.0047 (7)	0.0114 (7)
C4B	0.0254 (9)	0.0307 (9)	0.0204 (9)	0.0003 (7)	0.0069 (7)	0.0102 (8)
C5A	0.0278 (9)	0.0187 (8)	0.0214 (9)	−0.0011 (7)	0.0017 (7)	0.0067 (7)
C5B	0.0237 (8)	0.0226 (9)	0.0225 (9)	0.0005 (7)	0.0031 (7)	0.0071 (7)
C6A	0.0242 (8)	0.0216 (8)	0.0173 (8)	0.0006 (7)	0.0051 (7)	0.0034 (7)
C6B	0.0247 (9)	0.0265 (9)	0.0167 (8)	0.0024 (7)	0.0055 (7)	0.0034 (7)
C11A	0.0265 (9)	0.0220 (9)	0.0160 (8)	0.0018 (7)	0.0054 (7)	0.0057 (7)
C11B	0.0266 (9)	0.0254 (9)	0.0198 (9)	0.0039 (7)	0.0072 (7)	0.0061 (7)
O5W	0.0327 (7)	0.0281 (7)	0.0284 (7)	−0.0004 (5)	0.0101 (5)	0.0053 (6)
O6W	0.0316 (7)	0.0293 (7)	0.0319 (7)	0.0014 (5)	0.0073 (6)	0.0130 (6)
O7W	0.0530 (9)	0.0423 (8)	0.0308 (8)	0.0173 (7)	0.0087 (7)	0.0097 (7)
O8W	0.1022 (14)	0.0273 (8)	0.0264 (8)	0.0089 (8)	0.0032 (8)	0.0064 (6)

### Geometric parameters (Å, º)

**Table:**

Mg1—O1W	2.0929 (14)	O6W—H62W	0.8600
Mg1—O2W	2.0732 (13)	O7W—H72W	0.8600
Mg1—O3W	2.1024 (14)	O7W—H71W	0.8900
Mg1—O4W	2.0804 (13)	O8W—H81W	0.9000
Mg1—O11A	2.0304 (13)	O8W—H82W	0.8900
Mg1—O11B	2.0237 (13)	N3A—C3A	1.471 (2)
O11A—C11A	1.254 (2)	N3B—C3B	1.469 (2)
O11B—C11B	1.253 (2)	N5A—C5A	1.476 (2)
O12A—C11A	1.256 (2)	N5B—C5B	1.470 (2)
O12B—C11B	1.258 (2)	C1A—C2A	1.387 (2)
O31A—N3A	1.220 (2)	C1A—C6A	1.388 (2)
O31B—N3B	1.219 (2)	C1A—C11A	1.510 (2)
O32A—N3A	1.223 (2)	C1B—C6B	1.391 (3)
O32B—N3B	1.228 (2)	C1B—C11B	1.514 (2)
O51A—N5A	1.222 (2)	C1B—C2B	1.389 (2)
O51B—N5B	1.224 (2)	C2A—C3A	1.384 (2)
O52A—N5A	1.221 (2)	C2B—C3B	1.383 (2)
O52B—N5B	1.227 (2)	C3A—C4A	1.382 (3)
O1W—H11W	0.9100	C3B—C4B	1.382 (3)
O1W—H12W	0.8800	C4A—C5A	1.383 (2)
O2W—H21W	0.7600	C4B—C5B	1.380 (2)
O2W—H22W	0.8700	C5A—C6A	1.380 (2)
O3W—H32W	0.9000	C5B—C6B	1.384 (2)
O3W—H31W	0.8000	C2A—H2A	0.9300
O4W—H41W	0.8000	C2B—H2B	0.9300
O4W—H42W	0.8300	C4A—H4A	0.9300
O5W—H51W	0.8600	C4B—H4B	0.9300
O5W—H52W	0.8600	C6A—H6A	0.9300
O6W—H61W	0.8600	C6B—H6B	0.9300
			
Mg1···H52W	3.2400	O12B···H2B	2.5200
Mg1···H62W^i^	3.2400	O12B···H41W	2.0000
O1W···O2W	3.0289 (18)	O12B···H71W^vi^	1.8700
O1W···O4W	2.8669 (18)	O31A···H4B^x^	2.5900
O1W···O5W	2.9702 (19)	O31A···H2A	2.4900
O1W···O8W	2.700 (2)	O31B···H2B	2.4800
O1W···O11A	2.9371 (18)	O31B···H2B^xi^	2.8400
O1W···O11B	2.9163 (18)	O32A···H81W^vii^	2.8800
O1W···O6W^ii^	2.7934 (19)	O32A···H4A	2.5200
O2W···O12A	2.8001 (18)	O32A···H82W^vii^	2.5400
O2W···O1W	3.0289 (18)	O32B···H4B	2.4900
O2W···O3W	2.9285 (18)	O32B···H72W^viii^	2.5000
O2W···O6W	2.7375 (18)	O51A···H82W^iv^	2.8300
O2W···O11A	2.8859 (17)	O51A···H4A^xiv^	2.5100
O2W···O11B	2.8740 (17)	O51A···H2B^iv^	2.8100
O2W···C11A	3.150 (2)	O51A···H4A	2.4600
O2W···O6W^i^	3.1813 (19)	O51B···H4B	2.4600
O3W···O11A	2.9108 (18)	O51B···H4B^xii^	2.7600
O3W···O6W^i^	2.9522 (19)	O52A···H82W^iv^	2.4500
O3W···O2W	2.9285 (18)	O52A···H6A	2.4200
O3W···O4W	2.9804 (18)	O52B···H62W	2.8500
O3W···O5W^iii^	2.7722 (18)	O52B···H6B	2.4700
O3W···O7W	2.8213 (19)	O52B···H31W^i^	2.8000
O3W···O52B^i^	3.093 (2)	O52B···H72W^i^	2.7900
O3W···O11B	2.9054 (18)	N3A···O31A^ix^	2.951 (2)
O4W···O12B	2.7310 (18)	N3A···O32B^x^	2.923 (2)
O4W···O5W	3.1235 (19)	N3B···O31B^xi^	3.194 (2)
O4W···C11B	3.163 (2)	N5A···O12B^iv^	2.897 (2)
O4W···O1W	2.8669 (18)	N5A···O31B^vii^	2.765 (2)
O4W···O3W	2.9804 (18)	N5B···O31A^ii^	3.134 (2)
O4W···O5W^iv^	2.7986 (18)	C1A···O51B^i^	3.201 (2)
O4W···O11A	2.9144 (17)	C2A···O52B^ii^	3.290 (2)
O4W···O11B	2.9336 (17)	C2A···O51B^i^	3.208 (2)
O5W···O1W	2.9702 (19)	C2B···O51A^iv^	3.130 (2)
O5W···O4W	3.1235 (19)	C3A···O32B^x^	3.095 (2)
O5W···O7W^iv^	2.9449 (19)	C4A···O32B^x^	3.167 (2)
O5W···O4W^iv^	2.7986 (18)	C4A···O51A^xiv^	3.416 (2)
O5W···O3W^v^	2.7722 (18)	C4A···O31B^vii^	3.183 (2)
O6W···O12A^ii^	2.8404 (19)	C4B···O32A^xiii^	3.349 (3)
O6W···O2W^i^	3.1813 (19)	C4B···O31A^xiii^	3.348 (2)
O6W···O3W^i^	2.9522 (19)	C5A···O12B^iv^	3.189 (2)
O6W···O2W	2.7375 (18)	C5A···O31B^vii^	2.981 (2)
O6W···C11A^ii^	3.336 (2)	C6A···O7W	3.389 (2)
O6W···O1W^ii^	2.7934 (19)	C11A···O6W^ii^	3.336 (2)
O7W···O52B^i^	3.212 (2)	C11A···O51B^i^	3.343 (2)
O7W···C6A	3.389 (2)	C6A···H41W^iv^	3.0900
O7W···O5W^iv^	2.9449 (19)	C11A···H61W^ii^	2.6400
O7W···O3W	2.8213 (19)	C11A···H21W	2.6600
O7W···O12B^vi^	2.708 (2)	C11B···H41W	2.6400
O7W···O32B^vii^	3.236 (2)	C11B···H71W^vi^	2.9700
O8W···O12A^ii^	2.7737 (19)	H2A···O31A	2.4900
O8W···O52A^iv^	2.968 (2)	H2A···O12A	2.5200
O8W···O32A^viii^	3.094 (2)	H2B···O51A^iv^	2.8100
O8W···O1W	2.700 (2)	H2B···O12B	2.5200
O11A···O1W	2.9371 (18)	H2B···O31B	2.4800
O11A···O3W	2.9108 (18)	H2B···O31B^xi^	2.8400
O11A···O4W	2.9144 (17)	H4A···O51A^xiv^	2.5100
O11A···O2W	2.8859 (17)	H4A···O32A	2.5200
O11B···O3W	2.9054 (18)	H4A···O51A	2.4600
O11B···O2W	2.8740 (17)	H4B···O31A^xiii^	2.5900
O11B···O4W	2.9336 (17)	H4B···O51B^xii^	2.7600
O11B···O1W	2.9163 (18)	H4B···O32B	2.4900
O12A···O6W^ii^	2.8404 (19)	H4B···O51B	2.4600
O12A···O2W	2.8001 (18)	H6A···O52A	2.4200
O12A···O8W^ii^	2.7737 (19)	H6A···H51W^iv^	2.5900
O12B···N5A^iv^	2.897 (2)	H6A···O11A	2.4500
O12B···O51A^iv^	3.164 (2)	H6A···O4W^iv^	2.8300
O12B···C5A^iv^	3.189 (2)	H6B···O52B	2.4700
O12B···O7W^vi^	2.708 (2)	H6B···O11B	2.4600
O12B···O52A^iv^	3.187 (2)	H11W···O8W	1.7900
O12B···O4W	2.7310 (18)	H11W···H82W	2.3300
O31A···N3A^ix^	2.951 (2)	H11W···H81W	2.4500
O31A···O32B^x^	3.218 (2)	H12W···H52W	2.3700
O31A···C4B^x^	3.348 (2)	H12W···H62W^ii^	2.3100
O31A···O31A^ix^	2.8352 (19)	H12W···O6W^ii^	1.9300
O31A···N5B^ii^	3.134 (2)	H12W···H61W^ii^	2.2100
O31A···O51B^ii^	3.036 (2)	H21W···O12A	2.1100
O31B···O31B^xi^	2.6950 (19)	H21W···C11A	2.6600
O31B···C5A^viii^	2.981 (2)	H22W···H61W	2.4300
O31B···O51A^viii^	2.949 (2)	H22W···H62W	2.4400
O31B···N5A^viii^	2.765 (2)	H22W···O6W	1.8700
O31B···O52A^viii^	3.198 (2)	H31W···O52B^i^	2.8000
O31B···N3B^xi^	3.194 (2)	H31W···H72W	2.4800
O31B···C4A^viii^	3.183 (2)	H31W···O7W	2.0200
O32A···C4B^x^	3.349 (3)	H31W···H62W^i^	2.5900
O32A···O8W^vii^	3.094 (2)	H32W···H52W^iii^	2.3300
O32B···O51B^xii^	2.972 (2)	H32W···H51W^iii^	2.3900
O32B···N3A^xiii^	2.923 (2)	H32W···H62W^i^	2.3900
O32B···O7W^viii^	3.236 (2)	H32W···O5W^iii^	1.8900
O32B···O31A^xiii^	3.218 (2)	H41W···O12B	2.0000
O32B···C4A^xiii^	3.167 (2)	H41W···C11B	2.6400
O32B···C3A^xiii^	3.095 (2)	H41W···C6A^iv^	3.0900
O51A···O12B^iv^	3.164 (2)	H42W···H52W^iv^	2.4200
O51A···C2B^iv^	3.130 (2)	H42W···O5W^iv^	1.9700
O51A···C4A^xiv^	3.416 (2)	H42W···H51W^iv^	2.3800
O51A···O31B^vii^	2.949 (2)	H51W···H42W^iv^	2.3800
O51B···O31A^ii^	3.036 (2)	H51W···O7W^iv^	2.1100
O51B···O32B^xii^	2.972 (2)	H51W···H71W^iv^	2.2900
O51B···C11A^i^	3.343 (2)	H51W···H6A^iv^	2.5900
O51B···C2A^i^	3.208 (2)	H51W···H32W^v^	2.3900
O51B···C1A^i^	3.201 (2)	H52W···H12W	2.3700
O52A···O8W^iv^	2.968 (2)	H52W···Mg1	3.2400
O52A···O12B^iv^	3.187 (2)	H52W···O1W	2.1700
O52A···O31B^vii^	3.198 (2)	H52W···H32W^v^	2.3300
O52B···C2A^ii^	3.290 (2)	H52W···H42W^iv^	2.4200
O52B···O3W^i^	3.093 (2)	H52W···O4W	2.5300
O52B···O7W^i^	3.212 (2)	H61W···O12A^ii^	2.0000
O1W···H52W	2.1700	H61W···C11A^ii^	2.6400
O2W···H62W^i^	2.6200	H61W···H12W^ii^	2.2100
O3W···H62W^i^	2.1400	H61W···H22W	2.4300
O4W···H6A^iv^	2.8300	H62W···H31W^i^	2.5900
O4W···H52W	2.5300	H62W···H22W	2.4400
O5W···H42W^iv^	1.9700	H62W···O52B	2.8500
O5W···H32W^v^	1.8900	H62W···Mg1^i^	3.2400
O6W···H12W^ii^	1.9300	H62W···O2W^i^	2.6200
O6W···H22W	1.8700	H62W···O3W^i^	2.1400
O7W···H31W	2.0200	H62W···H12W^ii^	2.3100
O7W···H51W^iv^	2.1100	H62W···H32W^i^	2.3900
O8W···H11W	1.7900	H71W···O12B^vi^	1.8700
O11A···H21W	2.6800	H71W···C11B^vi^	2.9700
O11A···H31W	2.8600	H71W···H51W^iv^	2.2900
O11A···H6A	2.4500	H72W···H31W	2.4800
O11B···H41W	2.7100	H72W···O52B^i^	2.7900
O11B···H22W	2.9000	H72W···O32B^vii^	2.5000
O11B···H32W	2.8500	H81W···O32A^viii^	2.8800
O11B···H6B	2.4600	H81W···H11W	2.4500
O11B···H11W	2.8700	H81W···O12A^ii^	1.9900
O12A···H21W	2.1100	H82W···O32A^viii^	2.5400
O12A···H61W^ii^	2.0000	H82W···H11W	2.3300
O12A···H2A	2.5200	H82W···O51A^iv^	2.8300
O12A···H81W^ii^	1.9900	H82W···O52A^iv^	2.4500
			
O1W—Mg1—O2W	93.28 (6)	O51B—N5B—O52B	123.98 (16)
O1W—Mg1—O3W	177.64 (6)	C6A—C1A—C11A	119.51 (14)
O1W—Mg1—O4W	86.78 (6)	C2A—C1A—C6A	120.12 (15)
O1W—Mg1—O11A	90.83 (6)	C2A—C1A—C11A	120.35 (15)
O1W—Mg1—O11B	90.20 (6)	C2B—C1B—C11B	120.41 (16)
O2W—Mg1—O3W	89.06 (6)	C6B—C1B—C11B	119.53 (14)
O2W—Mg1—O4W	179.66 (6)	C2B—C1B—C6B	120.04 (15)
O2W—Mg1—O11A	89.38 (5)	C1A—C2A—C3A	118.30 (15)
O2W—Mg1—O11B	89.09 (5)	C1B—C2B—C3B	118.44 (16)
O3W—Mg1—O4W	90.88 (6)	N3A—C3A—C4A	118.58 (15)
O3W—Mg1—O11A	89.53 (6)	N3A—C3A—C2A	117.81 (15)
O3W—Mg1—O11B	89.50 (6)	C2A—C3A—C4A	123.62 (15)
O4W—Mg1—O11A	90.29 (5)	N3B—C3B—C2B	118.31 (15)
O4W—Mg1—O11B	91.24 (5)	N3B—C3B—C4B	118.16 (15)
O11A—Mg1—O11B	178.20 (6)	C2B—C3B—C4B	123.52 (15)
Mg1—O11A—C11A	134.86 (11)	C3A—C4A—C5A	115.94 (16)
Mg1—O11B—C11B	133.81 (12)	C3B—C4B—C5B	116.09 (16)
H11W—O1W—H12W	103.00	C4A—C5A—C6A	122.98 (16)
Mg1—O1W—H11W	121.00	N5A—C5A—C4A	118.66 (15)
Mg1—O1W—H12W	122.00	N5A—C5A—C6A	118.35 (15)
Mg1—O2W—H22W	121.00	N5B—C5B—C6B	118.97 (15)
H21W—O2W—H22W	104.00	C4B—C5B—C6B	123.07 (17)
Mg1—O2W—H21W	108.00	N5B—C5B—C4B	117.96 (15)
Mg1—O3W—H31W	122.00	C1A—C6A—C5A	119.03 (15)
Mg1—O3W—H32W	113.00	C1B—C6B—C5B	118.81 (15)
H31W—O3W—H32W	108.00	O11A—C11A—O12A	125.65 (15)
Mg1—O4W—H41W	109.00	O11A—C11A—C1A	116.26 (15)
Mg1—O4W—H42W	116.00	O12A—C11A—C1A	118.09 (14)
H41W—O4W—H42W	105.00	O12B—C11B—C1B	117.76 (15)
H51W—O5W—H52W	110.00	O11B—C11B—O12B	125.84 (16)
H61W—O6W—H62W	110.00	O11B—C11B—C1B	116.38 (15)
H71W—O7W—H72W	105.00	C3A—C2A—H2A	121.00
H81W—O8W—H82W	112.00	C1A—C2A—H2A	121.00
O32A—N3A—C3A	117.58 (16)	C3B—C2B—H2B	121.00
O31A—N3A—O32A	124.23 (16)	C1B—C2B—H2B	121.00
O31A—N3A—C3A	118.19 (14)	C3A—C4A—H4A	122.00
O31B—N3B—C3B	118.03 (14)	C5A—C4A—H4A	122.00
O32B—N3B—C3B	118.34 (15)	C3B—C4B—H4B	122.00
O31B—N3B—O32B	123.62 (15)	C5B—C4B—H4B	122.00
O51A—N5A—C5A	117.93 (15)	C5A—C6A—H6A	120.00
O52A—N5A—C5A	117.70 (15)	C1A—C6A—H6A	120.00
O51A—N5A—O52A	124.36 (16)	C1B—C6B—H6B	121.00
O52B—N5B—C5B	117.88 (15)	C5B—C6B—H6B	121.00
O51B—N5B—C5B	118.14 (15)		
			
O1W—Mg1—O11A—C11A	−76.77 (16)	C11A—C1A—C6A—C5A	177.32 (14)
O2W—Mg1—O11A—C11A	16.50 (16)	C6A—C1A—C11A—O11A	3.6 (2)
O3W—Mg1—O11A—C11A	105.57 (16)	C2A—C1A—C11A—O12A	1.7 (2)
O4W—Mg1—O11A—C11A	−163.55 (16)	C2A—C1A—C6A—C5A	−1.1 (2)
O1W—Mg1—O11B—C11B	−84.11 (16)	C11A—C1A—C2A—C3A	−177.50 (14)
O2W—Mg1—O11B—C11B	−177.38 (16)	C6A—C1A—C2A—C3A	0.9 (2)
O3W—Mg1—O11B—C11B	93.55 (16)	C2B—C1B—C6B—C5B	−1.6 (2)
O4W—Mg1—O11B—C11B	2.68 (16)	C6B—C1B—C2B—C3B	1.3 (2)
Mg1—O11A—C11A—O12A	9.1 (3)	C11B—C1B—C2B—C3B	179.69 (14)
Mg1—O11A—C11A—C1A	−171.22 (11)	C6B—C1B—C11B—O11B	−2.7 (2)
Mg1—O11B—C11B—O12B	−12.6 (3)	C2B—C1B—C11B—O12B	−2.6 (2)
Mg1—O11B—C11B—C1B	165.81 (11)	C11B—C1B—C6B—C5B	−179.97 (13)
O31A—N3A—C3A—C4A	−154.62 (15)	C6B—C1B—C11B—O12B	175.83 (15)
O32A—N3A—C3A—C4A	25.5 (2)	C2B—C1B—C11B—O11B	178.90 (14)
O31A—N3A—C3A—C2A	25.5 (2)	C1A—C2A—C3A—N3A	179.86 (14)
O32A—N3A—C3A—C2A	−154.31 (17)	C1A—C2A—C3A—C4A	0.0 (2)
O31B—N3B—C3B—C2B	21.9 (2)	C1B—C2B—C3B—N3B	−178.65 (13)
O32B—N3B—C3B—C2B	−159.03 (15)	C1B—C2B—C3B—C4B	0.2 (2)
O32B—N3B—C3B—C4B	22.0 (2)	C2A—C3A—C4A—C5A	−0.7 (2)
O31B—N3B—C3B—C4B	−156.99 (15)	N3A—C3A—C4A—C5A	179.43 (14)
O52A—N5A—C5A—C6A	11.0 (2)	N3B—C3B—C4B—C5B	177.46 (13)
O51A—N5A—C5A—C6A	−169.81 (15)	C2B—C3B—C4B—C5B	−1.4 (2)
O52A—N5A—C5A—C4A	−167.74 (15)	C3A—C4A—C5A—C6A	0.5 (2)
O51A—N5A—C5A—C4A	11.5 (2)	C3A—C4A—C5A—N5A	179.18 (14)
O52B—N5B—C5B—C6B	−17.5 (2)	C3B—C4B—C5B—N5B	−179.44 (13)
O51B—N5B—C5B—C6B	162.42 (15)	C3B—C4B—C5B—C6B	1.1 (2)
O51B—N5B—C5B—C4B	−17.0 (2)	N5A—C5A—C6A—C1A	−178.28 (14)
O52B—N5B—C5B—C4B	163.06 (15)	C4A—C5A—C6A—C1A	0.4 (2)
C6A—C1A—C11A—O12A	−176.77 (15)	N5B—C5B—C6B—C1B	−179.11 (14)
C2A—C1A—C11A—O11A	−178.01 (14)	C4B—C5B—C6B—C1B	0.3 (2)

Symmetry codes: (i) −*x*+2, −*y*+1, −*z*+1; (ii) −*x*+1, −*y*+1, −*z*+1; (iii) *x*+1, *y*, *z*; (iv) −*x*+1, −*y*, −*z*+1; (v) *x*−1, *y*, *z*; (vi) −*x*+2, −*y*, −*z*+1; (vii) *x*, *y*, *z*+1; (viii) *x*, *y*, *z*−1; (ix) −*x*+1, −*y*+1, −*z*+2; (x) *x*−1, *y*, *z*+1; (xi) −*x*+2, −*y*, −*z*; (xii) −*x*+2, −*y*+1, −*z*; (xiii) *x*+1, *y*, *z*−1; (xiv) −*x*+1, −*y*, −*z*+2.

### Hydrogen-bond geometry (Å, º)

**Table:**

*D*—H···*A*	*D*—H	H···*A*	*D*···*A*	*D*—H···*A*
O1*W*—H11*W*···O8*W*	0.91	1.79	2.700 (2)	179
O1*W*—H12*W*···O6*W*^ii^	0.88	1.93	2.7934 (19)	170
O2*W*—H21*W*···O12*A*	0.76	2.11	2.8001 (18)	152
O2*W*—H22*W*···O6*W*	0.87	1.87	2.7375 (18)	178
O3*W*—H31*W*···O7*W*	0.80	2.02	2.8213 (19)	170
O3*W*—H32*W*···O5*W*^iii^	0.90	1.89	2.7722 (18)	170
O4*W*—H41*W*···O12*B*	0.80	2.00	2.7310 (18)	151
O4*W*—H42*W*···O5*W*^iv^	0.83	1.97	2.7986 (18)	174
O5*W*—H51*W*···O7*W*^iv^	0.86	2.11	2.9449 (19)	164
O5*W*—H52*W*···O1*W*	0.86	2.17	2.9702 (19)	155
O5*W*—H52*W*···O4*W*	0.86	2.53	3.1235 (19)	127
O6*W*—H61*W*···O12*A*^ii^	0.86	2.00	2.8404 (19)	163
O6*W*—H62*W*···O3*W*^i^	0.86	2.14	2.9522 (19)	159
O7*W*—H71*W*···O12*B*^vi^	0.89	1.87	2.708 (2)	158
O7*W*—H72*W*···O32*B*^vii^	0.86	2.50	3.236 (2)	145
O8*W*—H81*W*···O12*A*^ii^	0.90	1.99	2.7737 (19)	145
O8*W*—H82*W*···O32*A*^viii^	0.89	2.54	3.094 (2)	122
O8*W*—H82*W*···O52*A*^iv^	0.89	2.45	2.968 (2)	117
C4*A*—H4*A*···O51*A*^xiv^	0.93	2.51	3.416 (2)	166
C4*B*—H4*B*···O31*A*^xiii^	0.93	2.59	3.348 (2)	139

Symmetry codes: (i) −*x*+2, −*y*+1, −*z*+1; (ii) −*x*+1, −*y*+1, −*z*+1; (iii) *x*+1, *y*, *z*; (iv) −*x*+1, −*y*, −*z*+1; (vi) −*x*+2, −*y*, −*z*+1; (vii) *x*, *y*, *z*+1; (viii) *x*, *y*, *z*−1; (xiii) *x*+1, *y*, *z*−1; (xiv) −*x*+1, −*y*, −*z*+2.
